# Joint Optimization and Performance Analysis of Analog Shannon–Kotel’nikov Mapping for OFDM with Carrier Frequency Offset

**DOI:** 10.3390/e27080778

**Published:** 2025-07-23

**Authors:** Jingwen Lin, Qiwang Chen, Yu Hua, Chen Chen

**Affiliations:** Xiamen Key Laboratory of Mobile Multimedia Communications, College of Information Science and Engineering, Huaqiao University, Xiamen 361021, China; linjingwen@stu.hqu.edu.cn (J.L.); yu.hua@stu.hqu.edu.cn (Y.H.); chen_chen@hqu.edu.cn (C.C.)

**Keywords:** analog JSCC, S-K mapping, OFDM, CFO, PAPR

## Abstract

An analog joint source-channel coding (AJSCC) based on Shannon–Kotel’nikov (S-K) mapping transmitting discrete-time encoded samples in orthogonal frequency division multiplexing (OFDM) systems over wireless channel has exhibited excellent performance. However, the phenomenon of carrier frequency offset (CFO) caused by the frequency mismatch between the transmitter’s and receiver’s local oscillators often exists in actual scenarios; thus, in this paper the performance of AJSCC-OFDM with CFO is analyzed and the S-K mapping is optimized. A joint optimization strategy is developed to maximize the signal-to-distortion ratio (SDR) subject to CFO constraints. Considering that the optimized AJSCC-OFDM strategies will change the amplitude distribution of encoded symbol, the peak-to-average power ratio (PAPR) characteristics under different AJSCC parameters are also analyzed.

## 1. Introduction

The separated coding system [1] initially eliminates redundant information via source coding techniques (e.g., quantization, entropy coding) and subsequently employs channel coding methods (e.g., low-density parity-check codes [2]) to introduce redundancy for enhancing interference resistance. While this system can theoretically achieve the Shannon limit under ideal assumptions [3], practical applications reveal several limitations; for example, the cascading error propagation caused by channel errors leads to the collapse of source decoding, the fixed redundant design in a low-noise environment results in low resource efficiency, and the infinite code length can not conform to actual communication. These limitations affect the adaptability of time-varying channels. On the other hand, traditional digital joint source-channel coding (JSCC) systems [4,5] aim to minimize end-to-end distortion by designing joint codebooks through the simultaneous optimization of source compression and channel protection at the coding level, which can approximate the Shannon limit. However, this approach encounters challenges such as explosive complexity, insufficient flexibility, and implementation difficulties. Consequently, these systems struggle to effectively handle real-time fading channel environments.

Shannon and Goblick [6,7] have proposed mapping information symbols onto parametric curves within the continuous signal space to approximate optimal performance limits using the geometric structure of high-dimensional signal spaces. Recent studies [8,9] indicate that the analog joint source channel coding (AJSCC) strategy achieves near-optimal performance through simple nonlinear symbol mapping. Specifically, this method directly maps source samples with continuous amplitudes to channel symbols to bypass the digitization process, including quantization, entropy coding, and error correction coding, while utilizing nonlinear curves or surfaces such as spiral curves for dimensionality transformation. The Shannon–Kotel’nikov (S-K) mapping-based AJSCC strategy combines continuous geometric structures with parameter adaptation, achieving performance close to the theoretical limit while maintaining low complexity [9]. The S-K mapping can realize direct source-to-channel mapping through spatial parameter curves and adopt finite parameters to shape the distribution of sending symbols, which is efficient and flexible enough to adapt to different communication scenarios. This strategy is particularly well suited for low-latency requirements in time-varying communication environments such as Internet of Things devices and real-time media transmission domains. Its core innovation lies in replacing discrete codebooks with nonlinear curves and achieving “progressive optimization” through analog processing, thereby providing an efficient and near-optimal solution for low-latency communications.

Recent advancements have been made in the implementation of maximum likelihood (ML) [8] and minimum mean square error (MMSE) [10] receiving algorithms for S-K mapping at the receiver end. In wireless communication scenarios, research has demonstrated that S-K mapping significantly enhances system performance in diversity reception technologies [11]. Multiple access technology [12] can increase the throughput of AJSCC systems. Combined with code-division multiple access (CDMA) [13], S-K mapping can realize multi-user analog joint transmission with low complexity and low delay in multi-access channels. Similarly, S-K mapping exhibits robust performance in multiple-input multiple-output (MIMO) systems [14]. The proposed mapping can achieve high spectral efficiency and low complexity in MIMO orthogonal frequency division multiplexing (OFDM) systems via MMSE precoding and adaptive parameter optimization, and the resulting performance approaches the theoretical limit [15].

In practical scenarios using OFDM, such as optical communications [16], low-earth orbit satellite communications [17], aeronautical communications, and high-speed rail communications [18], unstable hardware components or high-speed terminal movement can often cause mismatches in the local oscillators between the transmitter and receiver, resulting in significant carrier frequency offset (CFO). This offset induces inter-carrier interference (ICI), posing serious challenges to multi-carrier communication system performance and necessitating in-depth research with effective solutions.

This paper investigates a joint optimization and performance analysis of the AJSCC-OFDM systems in the presence of CFO. Furthermore, considering that AJSCC parameter changes may affect the symbol distribution of the transmission channel, we examine the peak-to-average power ratio (PAPR) at the transmitter. Finally, we present the variations in PAPR for the AJSCC-OFDM systems.

The remainder of this paper is organized as follows: Section 2 reviews the fundamental knowledge of AJSCC-OFDM systems; Section 3 analyzes the impact of CFO on the AJSCC-OFDM systems; in Section 4, we discusses the joint optimization in the presence of CFO; the PAPR is analyzed in Section 5; Section 6 presents our simulation results and experimental findings; finally, Section 7 provides concluding remarks.

## 2. System Model

**Figure 1:**
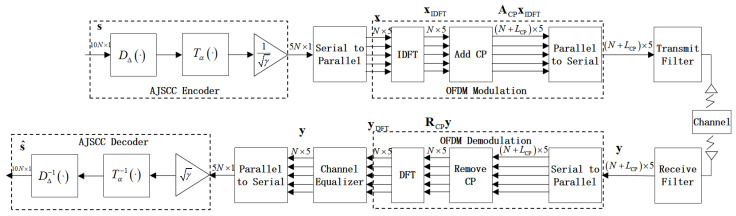
Block diagram of a bandwidth compression 2:1 AJSCC-OFDM systems over multiple fading channel.

In this section, we present the AJSCC strategy in OFDM systems over a multiple fading channel. Figure 1 shows the block diagram of a discrete-time AJSCC-OFDM systems.

### 2.1. AJSCC-OFDM Transmitter

At the source end, source symbols are assembled into $s=\{s_{i},0,1,2,\cdots ,2N-1\}$, which are independent and identically distributed (i.i.d.) Gaussian random variables, then compressed into a channel symbol $x=\{x_{i},0,1,2,\cdots ,N-1\}$ to achieve bandwidth compression for the systems.

Encoding in AJSCC typically involves three steps: a compression function $D_{\Delta }$, a matching function $T_{\alpha }$, and a power normalization processing *R*. The compression function can employ S-K mapping, taking the case of 2:1 compression as an example, to map the source vector $z=(z_{0},z_{1})$ to a single value ψ. This ψ value is constrained to lie on spiral-like curves, as provided by

(1) $$z_{\Delta }\left(\psi \right)=\left(\frac{\Delta }{\pi }\psi \cos\left(\psi \right),\frac{\Delta }{\pi }\left|\psi \right|\sin\left(\psi \right)\right),$$

where Δ is the distance between two neighboring spiral arms and ψ is the angle from the origin to the point $z=(z_{0},z_{1})$ on the curve. A larger Δ implies greater noise tolerance on the part of the mapping scheme, which is suitable for information transmission with low signal-to-noise ratios (SNRs). On the contrary, in the case of large SNR the influence of channel noise can be ignored, and it is more appropriate to adopt the coding curve with small Δ. Given a specific spiral defined by its Δ value, the compression function $D_{\Delta }$ calculates the value $\hat{\psi }$ corresponding to the point on the spiral that minimizes the distance to the point s, i.e.,

(2) $$\hat{\psi }=D_{\Delta }\left(s\right)={\mathrm{argmin}\|s-z_{\Delta }\left(\psi \right)\|}^{2}.$$

Then, the matching function is used to control the distribution of transmitted symbols in order to adapt to the characteristics of different channels, which can be written as

(3) $$T_{\alpha }\left(\psi \right)=\mathrm{sign}\left(\psi \right){\left|\psi \right|}^{\alpha },$$

where α∈(0,2]. We can flexibly adjust the value of α so that the systems match the time-varying characteristics of the fading channel.

Lastly, we need to ensure that the average transmitted power is equal to one; thus, the signal symbol is provided by

(4) $$x=R\left(s\right)=\frac{T_{\alpha }\left(D_{\Delta }\left(s\right)\right)}{\sqrt{\gamma }},$$

where γ is the mean square energy of the transmitted symbol $x\in R^{N\times 1}$ such that $E[|x{|}^{2}]=1$ and E[.] is the expectation operator representing the statistical average of the random variables.

The encoded symbol vector x in the frequency domain is modulated by the inverse discrete Fourier transform (IDFT) to generate a time-domain signal $x_{\mathrm{IDFT}}\in C^{N\times 1}$, i.e.,

(5) $$x_{\mathrm{IDFT}}=F^{H}x,$$

where F denotes the normalized DFT matrix and ${\left(.\right)}^{H}$ denotes the complex conjugate transpose of vectors and matrices.

### 2.2. Input–Output Relation

Next, we take into account the complex channel case involving multipath fading channels. To avoid inter-block interference (IBI) from multipath fading channels, the OFDM modulator usually adds a cyclic prefix (CP) for each OFDM symbol $x_{\mathrm{IDFT}}$ via the CP addition matrix $A_{\mathrm{CP}}$, with $N_{\mathrm{CP}}$ being the length of the CP. Hence, a CP-OFDM block of length $N+L_{\mathrm{CP}}$, denoted by $x_{\mathrm{CP}}$, is created as

(6) $$x_{\mathrm{CP}}=A_{\mathrm{CP}}x_{\mathrm{IDFT}}={\left[\begin{array}{cc} I_{\mathrm{CP}}^{T} & I_{N} \end{array}\right]}^{T}x_{\mathrm{IDFT}},$$

where $I_{\mathrm{CP}}$ is the last $L_{\mathrm{CP}}$ rows of $I_{N}$ and $A_{\mathrm{CP}}$ is of size $(N+L_{\mathrm{CP}})\times N$. We assume that the parameter $L_{\mathrm{CP}}$ is no less than the maximum delay spread of the channel, which is denoted by *L*. We have $L_{\mathrm{CP}}\geq L$; thus, the IBI can be eliminated.

The transmitted signal passes through a multipath channel. The channel is static or quasi-static, which means that the channel response remains constant within one OFDM block and might change in the next block. Thus, the received CP-OFDM block of length $N+L_{\mathrm{CP}}$ can be represented as

(7) $$y_{\mathrm{CP}}=H_{0}x_{\mathrm{CP}}+H_{1}^{\prime }x_{\mathrm{CP}}^{\prime }+w,$$

where $H_{0}$ is a lower triangular Toeplitz matrix with its first column as $h_{0}=[h\left(0\right),h\left(1\right),\ldots ,$${h\left(L-1\right),0,\ldots ,0]}^{T}$, while $H_{1}^{\prime }$ is an upper triangular Toeplitz matrix with its first column as $h_{1}^{\prime }={\left[0,\ldots ,0,h^{\prime }\left(L-1\right),\ldots ,h^{\prime }\left(0\right)\right]}^{T}$. Here, $x_{\mathrm{CP}}^{\prime }$ denotes the previous symbol block, while $h_{1}^{\prime }$ denotes the channel fading coefficient undergone by symbol block $x_{\mathrm{CP}}^{\prime }$ and w∼$\mathrm{CN}(0,\sigma^{2}I)$ is the AWGN vector.

By removing the CP of $x_{\mathrm{CP}}$ at the receiver, the interference from the previous block is canceled out. Accordingly, the stripped symbol block, denoted by r of length *N*, is obtained and modeled as

(8) $$y_{R}=R_{\mathrm{CP}}y=R_{\mathrm{CP}}H_{0}A_{\mathrm{CP}}x_{\mathrm{IDFT}}+R_{\mathrm{CP}}w=Hx_{\mathrm{IDFT}}+R_{\mathrm{CP}}w,$$

where $R_{\mathrm{CP}}=\left[0_{N\times L_{\mathrm{CP}}}\;I_{N}\right]$ and H is a circulant channel impulse response (CIR) matrix for static multipath fading channels.

### 2.3. AJSCC-OFDM Receiver

After OFDM demodulation, the received signal is

(9) $$y_{\mathrm{DFT}}=F(Hx_{\mathrm{IDFT}}+R_{\mathrm{CP}}w)=\Lambda x+\tilde{w},$$

where $H=F\Lambda F^{H}$ with channel frequency response (CFR) matrix Λ and where $\tilde{w}≜FR_{\mathrm{CP}}w$.

In the case of the multipath fading channels, when the equivalent channel matrix Λ is known at the receiving end, the equalized signal is provided by

(10) $$y=Gy_{\mathrm{DFT}}=G\Lambda x+G\tilde{w},$$

where G is a diagonal matrix with its *k*-th diagonal entry G(k) as the coefficients of the single-tap equalizer. For example, if MMSE is adopted, then G(k) is

(11) $$G\left(k\right)=\frac{\Lambda^{*}\left(k\right)}{|\Lambda \left(k\right){|}^{2}+{\mathrm{SNR}}^{-1}},$$

where $\mathrm{SNR}={E[|x|}^{2}{]/E[|w|}^{2}]$.

When the MMSE equalizer is employed, the recovered signal y is provided by

(12) $$\begin{array}{c} y=\left(\frac{\Lambda^{H}\Lambda }{\Lambda^{H}\Lambda +{\mathrm{SNR}}^{-1}I}\right)x+\frac{\Lambda^{H}}{\Lambda^{H}\Lambda +{\mathrm{SNR}}^{-1}I}\tilde{w}. \end{array}$$

In practice, the MMSE equalizer efficiently balances channel compensation with noise suppression, improving the recovery accuracy of y.

At the receiving end, we perform a series of inverse transformations in turn: $R^{-1}$, $T_{\alpha }^{-1}$, $D_{\Delta }^{-1}$. The system performance is quantified by the signal-to-distortion ratio (SDR) versus the SNR:

(13) $$\mathrm{SDR}=10\log_{10}\left(\frac{E[|x{|}^{2}]}{\mathrm{MSE}}\right)$$

where the distortion is measured as the mean square error (MSE) between decoded and source signals:

(14) $$\mathrm{MSE}=\frac{1}{N}E\left\{∥s-\hat{s}{∥}^{2}\right\}.$$

## 3. Analysis of AJSCC-OFDM with Carrier Frequency Offset

In communication systems, high-speed motion of the transmitter and receiver (e.g., satellites, aircraft, high-speed trains) leads to signal frequency compression or stretching. Additionally, insufficient precision or temperature-induced drift in local oscillators at both the transmitter and receiver result in mismatches between carrier frequencies. Even a tiny frequency offset can disrupt the orthogonality of subcarriers [19] and induce ICI. In this section, the impact of CFO is taken into account in the optimization.

Assuming that the normalized CFO is ε, phase rotation is introduced into the received time domain signal due to frequency offset; thus, Equation (8) becomes

(15) $$y_{R}=CHx_{\mathrm{IDFT}}+R_{\mathrm{CP}}w,$$

where C is the diagonal matrix of N×N and the diagonal elements are $C_{n,n}=e^{j2\pi \varepsilon n/N}$. Time-domain analysis indicates that CFO will introduce phase rotation. To fully understand its influence, the frequency-domain distortion caused by CFO is analyzed below.

The receiver performs DFT on $y_{R}$ to obtain a signal in the frequency domain:

(16) $$\begin{array}{c} y_{\mathrm{DFT}}=Fy_{R}=FCF^{H}\Lambda x+\tilde{w}=I_{\mathrm{CFO}}\Lambda x+\tilde{w}, \end{array}$$

where $I_{\mathrm{CFO}}=FCF^{H}$ is the frequency domain interference matrix caused by the CFO.

The element (p,q) of matrix $I_{\mathrm{CFO}}$ represents the interference coefficient of the *q*-th subcarrier to the *p*-th subcarrier, namely,

(17) $$\begin{array}{c} I_{\mathrm{CFO}}\left(p,q\right)=\frac{1}{N}\sum_{n=0}^{N-1}e^{j2\pi (\varepsilon +q-p)n/N}=\frac{\sin(\pi (\varepsilon +q-p))}{N\sin\left(\frac{\pi (\varepsilon +q-p)}{N}\right)}e^{j\pi (\varepsilon +q-p)(N-1)/N}, \end{array}$$

which is basically the ICI matrix in OFDM systems impaired by CFO. As shown in [20], assuming that the receiver has ideal channel state information (CSI) and can fully compensate the total phase offset, the effective channel Λ can be transformed into $e^{\frac{j2\pi \epsilon (N+L_{\mathrm{CP}})}{N}}e^{\frac{j\pi \epsilon (N-1)}{N}}\Lambda$, which produces a time-varying rotation of the AJSCC-OFDM data.

Then, by considering the effective channel as expressed by $I_{\mathrm{CFO}}\Lambda$ and performing the classical zero-forcing equalization, from Equation (16) we obtain

(18) $$y=\Lambda^{-1}I_{\mathrm{CFO}}^{-1}y_{\mathrm{DFT}}=x+\Lambda^{-1}I_{\mathrm{CFO}}^{-1}\tilde{w},$$

where $I_{\mathrm{CFO}}^{-1}\left(p,q\right)=N\sum_{n=0}^{N-1}e^{-j2\pi (\varepsilon +q-p)n/N}$, which results in an increase in equivalent noise power and changes the noise distribution characteristics.

## 4. Joint Optimization

As established in the preceding section, increasing the AJSCC parameter Δ enhances the system’s noise immunity, while adaptive adjustment of the α parameter enables dynamic symbol distribution shaping to accommodate complex channel conditions. Therefore, we advocate re-optimizing the systems.

This section develops a joint (Δ,α) optimization strategy to maximize the SDR of AJSCC-OFDM transmissions subject to CFO constraints. The joint optimization strategy is summarized in Algorithm 1. During the simulation process, for each SNR condition, the AJSCC encoder adapts to different CFO factors ϵ in the fading channel by traversing its own parameters, as shown in the algorithm on line 6.
**Algorithm 1** Joint Optimization Scheme**Input:** SNR,s,N,ϵ   1:Initialize α=0.1, Δ=0.01   2:**for** α=0.1 to 2.0 **do**   3:   **for** Δ=0.01 to 3.00 **do**   4:       [x,γ]←AJSCC/encoder(s,α,Δ,SNR)   5:       $x_{\mathrm{IDFT}}\leftarrow OFDM/transmitter\left(x\right)$   6:       $\left[y_{R},\Lambda \right]\leftarrow Channel\left(x_{\mathrm{IDFT}},\mathrm{SNR},\epsilon \right)$   7:       $y_{\mathrm{DFT}}\leftarrow OFDM/receiver\left(y_{R}\right)$   8:       $y\leftarrow Channel/Equalizer(y_{\mathrm{DFT}},\mathrm{CSNR},\Lambda )$   9:       $\hat{s}\leftarrow AJSCC/decoder\left(y,\gamma \right)$ 10:   **end for** 11:**end for** 12:find the maximum SDR and record corresponding parameters: (Δ,α)**Output:** ${\mathrm{SDR}}_{\max},\Delta_{\mathrm{opt}},\alpha_{\mathrm{opt}}$

In AJSCC systems, the parameter Δ represents the double spiral arm spacing of the mapping curve, which directly determines the distribution characteristics of the coding symbol in the signal space. The value of the Δ parameter is positively correlated with the mapping distortion [21] and negatively correlated with system performance. With a larger Δ, the mapping curve is more sparse and the mapping error is larger; as a result, the system performance decreases. Specifically: (1) Relationship between the value of Δ and the spatial distribution: When the value of Δ is small, the mapping curve shows a dense distribution in the sign space, which can cover the source space more precisely and improve the encoding resolution, whereas when the value of Δ is large, the mapping curve distribution is relatively scattered and a large interval is maintained between each coding symbol.

(2) Trade-off between Δ and system robustness: In scenarios with poor channel conditions (high noise), adopting a larger Δ value can enhance the robustness of the systems to noise and reduce system distortion by increasing the symbol spacing; when the channel conditions are excellent (low noise), a smaller Δ value can be selected to reduce the mapping error through a dense spiral distribution.

(3) Practical application considerations: According to the real-time channel conditions, the systems adjust the key parameters Δ and α to cope with different degrees of carrier offset. This can be seen from the fluctuation of the data Δ in Table 1. When a significant CFO (equivalent noise power increase) is detected, the systems automatically increase the Δ value and enhance the noise tolerance by extending the symbol spacing. In harsh channel conditions (high CFO), a large Δ value combined with an optimized α parameter is used to ensure communication reliability. In this case, the systems gradually reduce Δ and adjust α to enhance system robustness as channel conditions improve.

## 5. Peak-to-Average Power Ratio

Given that joint optimization of (Δ,α) will change the amplitude distribution of emission symbol, in this section we present simulations carried out to investigate the PAPR characteristics of the proposed AJSCC-OFDM systems. Ignoring the CP, the instantaneous PAPR [22] is defined by

(19) $$\mathrm{PAPR}\left(\mathrm{dB}\right)=10\log_{10}\left(\frac{\max_{0\leq n\leq N-1}{\left|x_{\mathrm{IDFT}}\left[n\right]\right|}^{2}}{E\left[|x_{\mathrm{IDFT}}{\left[n\right]|}^{2}\right]}\right).$$

The PAPR is evaluated by complementary cumulative distribution function (CCDF), which is defined as the probability of the PAPR of a signal exceeding a threshold ${\mathrm{PAPR}}_{0}$, i.e.,

(20) $$\mathrm{CCDF}=\mathrm{Pr}[\mathrm{PAPR}>{\mathrm{PAPR}}_{0}].$$

Assuming that the AJSCC symbols are zero-mean and i.i.d. random variables with independent in-phase and quadrature components, the real and imaginary parts of the *l*-th element of an AJSCC-OFDM block are provided by

(21) Re{xIDFT[l]}=1N∑k=0N−1Re{x[k]}cos2πklN−Im{x[k]}sin2πklN,

(22) Im{xIDFT[l]}=1N∑k=0N−1Re{x[k]}sin2πklN+Im{x[k]}cos2πklN.

The real part is not correlated with the imaginary part when ERe{x[l]}·Im{x[l]}=ERe{x[l]}·EIm{x[l]}. Because X[k] is a Laplacian distribution with zero mean, it is obvious that ERe{x[l]}=EIm{x[l]}=0; thus, it is only necessary to prove

(23) ERe{x[l]}·Im{x[l]}=0.

The expectation of expanding the product is

**Figure 2:**
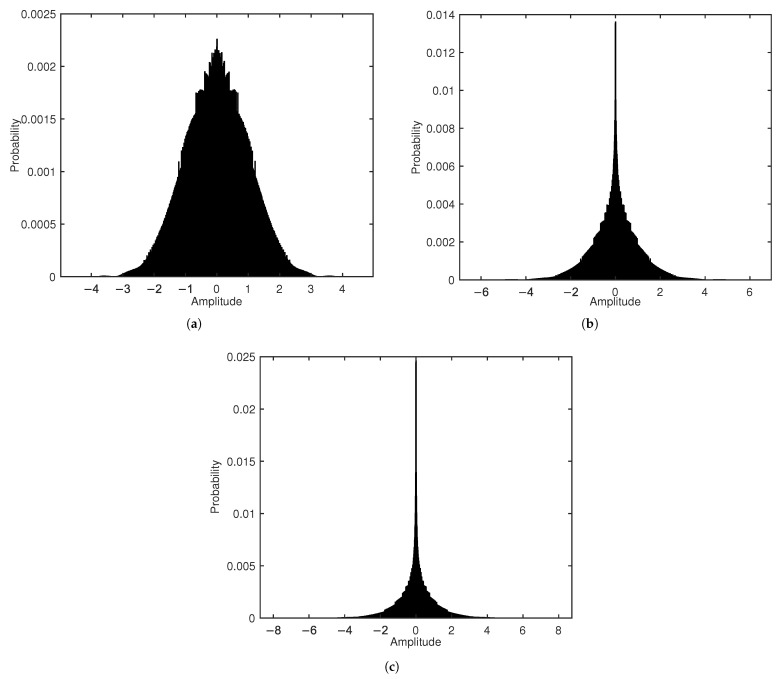
The amplitude probability distribution of x when Δ=1.50 with α=1.3, α=1.7 and α=2.0: (**a**) α=1.3, (**b**) α=1.7, (**c**) α=2.0.

(24) ERe{xIDFT[l]}·Im{xIDFT[l]}=1N2∑k=0N−1∑k′=0N−1E[Re{x[k]}cosθkl−Im{x[k]}sinθkl·Re{x[k′]}sinθk′l+Im{x[k′]}cosθk′l],

where $\theta_{kl}=\frac{2\pi kl}{N}$. Using the statistical properties of x[k], we have the following:

(1) Independence: Both x[k] and x[l] are independent (k≠l); therefore, we have cross-terms ERe{x[k]}Im{x[l]}=0.
(2) Zero mean: ERe{x[k]}=EIm{x[k]}=0.
(3) No correlation between real and imaginary parts: For further details, please refer to the symbol distribution presented in Figure 2, which adheres to the aforementioned three statistical characteristics. Let us say ERe{x[k]}Im{x[k]}=0; thus, we only need to consider the terms $k=k^{\prime }$:

(25) ERe{xIDFT[l]}·Im{xIDFT[l]}=1N2∑k=0N−1cosθklsinθklERe{x[k]}2−sinθklcosθklEIm{x[k]}2.

If ERe{x[k]}2=EIm{x[k]}2 (power balance), then the above formula simplifies to

(26) 1N2∑k=0N−1cosθklsinθkl−sinθklcosθklERe{x[k]}2=0.

Thus, the real and imaginary parts of the time-domain symbol x[n] satisfy Equation (24).

After establishing that x consists of i.i.d. random variables with zero mean, we can invoke Lyapunov’s central limit theorem to demonstrate that as *N* approaches infinity, the AJSCC-OFDM symbols can be approximated by i.i.d. normal random variables. Consequently, the CCDF of PAPR of AJSCC-OFDM changes from Equation (25) to the following formula:

(27) $$\begin{array}{c} \mathrm{Pr}\left[\mathrm{PAPR}>{\mathrm{PAPR}}_{0}\right]=1-{\left[1-e^{-{\mathrm{PAPR}}_{0}}\right]}^{N} \end{array}$$

which is identical to that of OFDM. Obviously, when the length of DFT increases, the value of CCDF for the same threshold ${\mathrm{PAPR}}_{0}$ will increase significantly.

## 6. Simulation

In this section, we focus on evaluating AJSCC-OFDM systems over multipath fading channels. Our analysis not only validates the preliminary findings but also extends the analysis through CFO and PAPR to verify the robustness of the AJSCC strategy in complex environments. A multipath channel model with strong interference, namely, a three-path channel with equal power gain, is considered to test the robustness of this strategy. Specifically, we consider a three-path channel L=3, i.e., $h_{0}={\left[h\left(0\right),h\left(1\right),h\left(2\right),0,\ldots ,0\right]}^{T}$, where h(.) obeys the normalized Rayleigh distribution and $E[{\left|h(.)\right|}^{2}]=1/3$.

### 6.1. Signal-to-Distortion Ratio Performance

**Figure 3:**
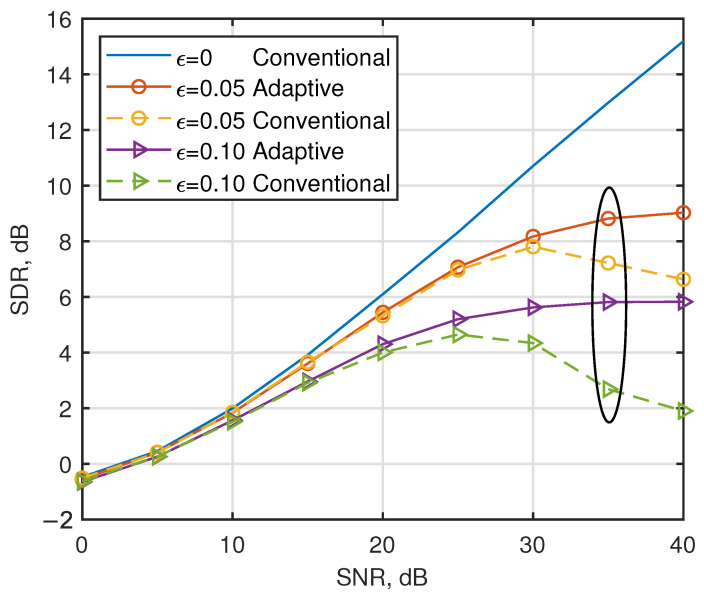
Performance comparison of the considered strategies in the presence of CFO with ϵ = 0, ϵ = 0.05, and ϵ = 0.10 over multipath fading channels.

In this subsection, the CFO is systematically analyzed for the frequency-sensitive characteristics of multi-carrier systems. Figure 3 shows the SDR for AJSCC-OFDM in the presence of CFO over multipath fading channels. Interestingly, the figure shows that the conventional parameters employed by [15] for multipath channels demonstrate limited robustness against phase offset induced by CFO, leading to performance degradation on the part of the systems. The proposed adaptive parameter optimization strategy effectively mitigates the SDR degradation observed in high-SNR regimes, achieving a 3.1 dB improvement compared to conventional parameter configurations when SNR = 35 dB with ϵ=0.10. Let us now turn our attention to the solid line. It can be seen that under the condition of low SNR (SNR < 15 dB), the noise power is dominant and the MMSE equalizer can effectively suppress the noise interference. However, in the case of high SNR (SNR = 35 dB), the influence of noise is negligible and the ICI caused by CFO becomes the main factor limiting system performance. The phase rotation caused by CFO not only leads to energy leakage between subcarriers due to ICI, but also disrupts the continuity of S-K mapping, preventing the accurate recovery of the analog signal during decoding at the receiving end. Specifically, when the normalized CFO factor ϵ increases from 0.05 to 0.10, the SDR of the AJSCC-OFDM systems decreases significantly by 3.0 dB.

**Figure 4:**
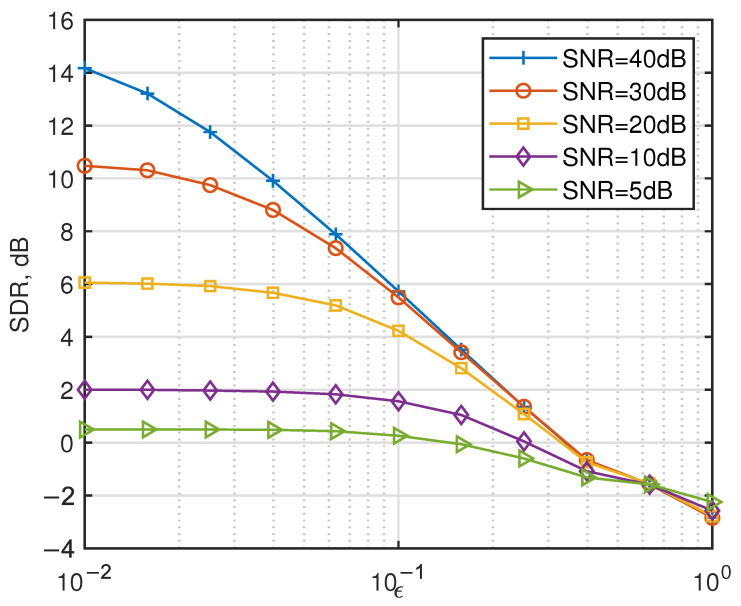
Performance of the considered strategies in the presence of CFO.

Figure 4 shows the SDR of the considered strategies for different ϵ. In high-SNR regimes, CFO-induced ICI becomes the dominant degradation factor. However, excessive CFO renders signal decoding infeasible irrespective of SNR levels.

**Figure 5:**
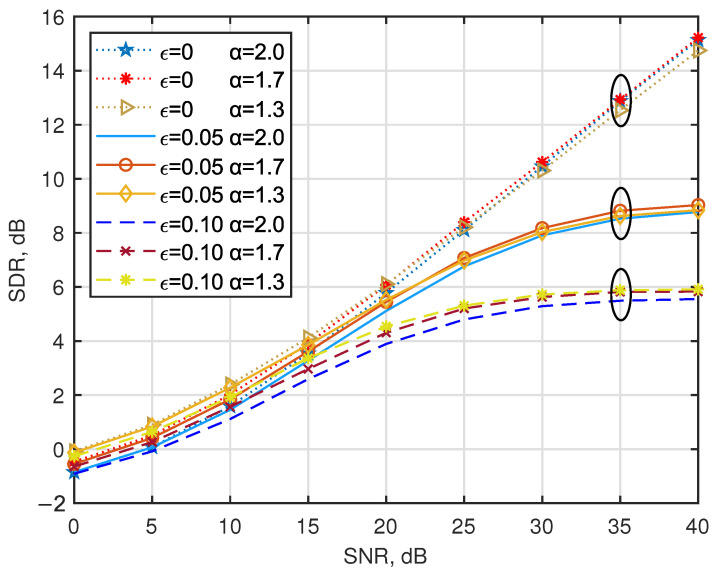
Performance comparison of the considered strategies in the presence of CFO with joint optimization of parameters α and Δ over multipath fading channels.

Figure 5 shows the SDR of AJSCC-OFDM in the presence of CFO for different AJSCC parameters α. The results demonstrate that α = 1.7 exhibits greater robustness against ICI-induced degradation compared to α = 2.0, which is consistently observed across both low and high SNR regimes. Moreover, the performance gap becomes larger with increasing frequency offset; when the offset factor ϵ rises from 0.05 to 0.10, the SDR difference expands from approximately 0.3 dB to 0.5 dB.

### 6.2. Peak-to-Average Power Ratio Performance

Considering the unique symbolic characteristics of AJSCC and the multi-carrier superposition effect in this subsection, the PAPR characteristics of the system transmitter are analyzed in detail.

**Figure 6:**
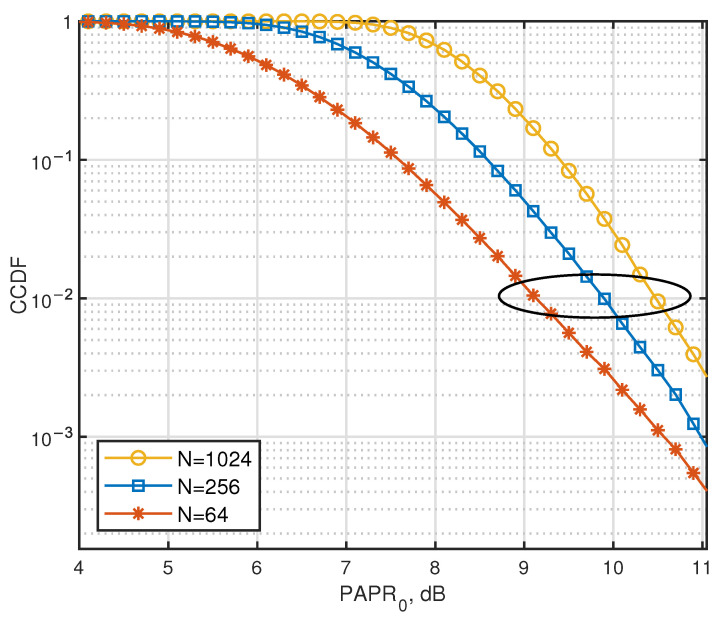
CCDF of PAPR for AJSCC-OFDM with different numbers of subcarriers.

Figure 6 provides the CCDF of the PAPR in AJSCC-OFDM systems. It can be seen that in analog communication, PAPR inevitably increases as the number of subcarriers *N* increases after the transmission waveform of multi-carrier systems is superimposed, as proved in the last part. The probability of PAPR exceeding 10.6 dB is less than $10^{-2}$ regardless of whether the number of subcarriers *N* is 64 or 1024.

**Figure 7:**
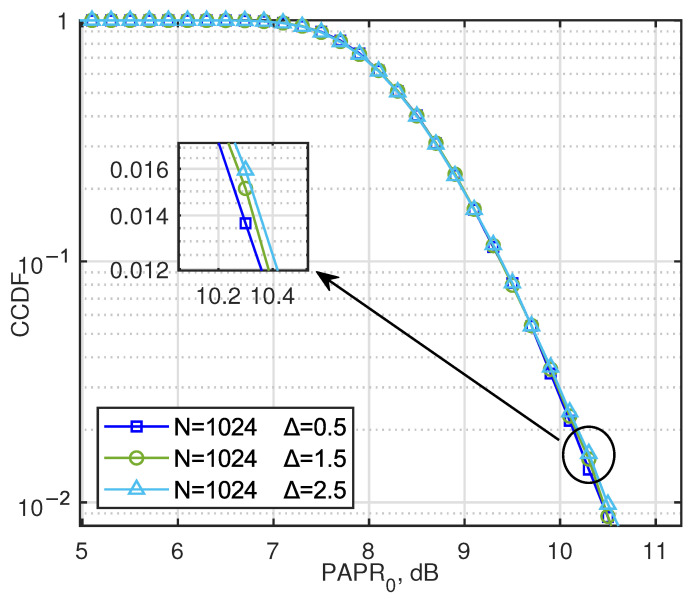
CCDF of PAPR for AJSCC-OFDM with the Δ values of AJSCC scheme set to 0.5, 1.5, and 2.5.

Further, the parameter optimization of AJSCC systems is considered, that is, the optimal delta is searched under each SNR. We provide the PAPR situation of the sender for different Δ cases, as shown in Figure 7. The results show that different Δ values have little effect on PAPR, which is mainly due to the normalization function of the AJSCC encoder.

**Figure 8:**
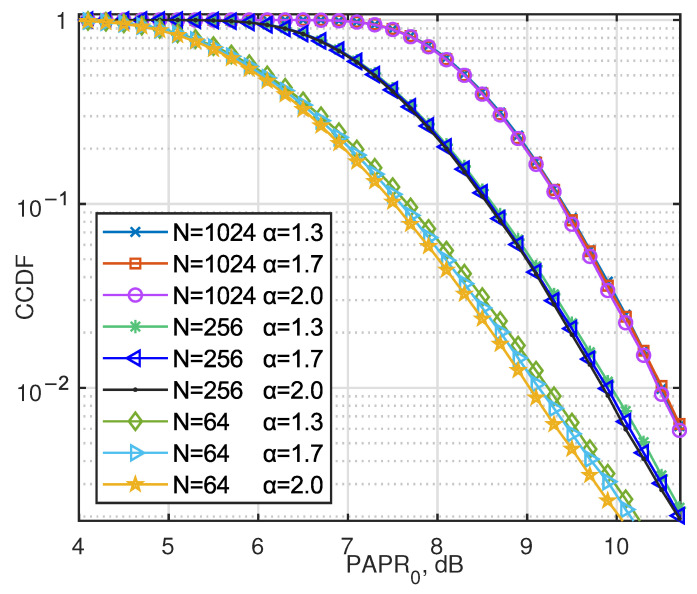
CCDF of PAPR for AJSCC-OFDM with the α values of AJSCC scheme set to 1.3, 1.7, and 2.0.

Figure 8 shows a significant correlation between the PAPR characteristics and the symbol distribution parameter α, especially when the number of subcarriers N=64. As shown in Figure 2, when α=2.0, the amplitude distribution of the symbols exhibits a high kurtosis characteristic with the energy concentrated near zero, resulting in a lower PAPR value; conversely, when α=1.3, the PAPR value is higher. However, as the length of the OFDM symbol block increases (i.e., the number of superimposed subcarriers increases), the central limit theorem effect gradually strengthens and the influence of the α parameter on PAPR gradually weakens.

## 7. Conclusions

In this paper, we have studied the effects of CFO for the AJSCC-OFDM systems over wireless channels. A joint parameter optimization strategy for the compression function (Δ) and matching function (α) is proposed to adaptively maximize the SDR. The simulation results indicate that when the SNR is high, the phase offset caused by CFO will significantly reduce system performance and that this deterioration degree will increase with the increase of the offset. Furthermore, our results show the AJSCC parameters maintain strong CFO resilience in multipath fading environments, demonstrating robust performance under realistic channel impairments. Lastly, this study shows that the AJSCC-OFDM parameter can reduce the PAPR at the transmitter.

## Figures and Tables

**Table 1:** The optimal Δ values under different SNR levels when ϵ=0, ϵ=0.05, and ϵ=0.10.

SNR (dB)	ϵ=0	ϵ=0.05	ϵ=0.10
0	2.92	2.94	2.96
5	2.89	2.90	2.94
10	2.65	2.71	2.93
15	2.13	2.18	2.30
20	1.70	1.82	2.04
25	1.24	1.41	1.98
30	0.98	1.39	1.90
35	0.73	1.30	1.78
40	0.62	1.28	1.91

## Data Availability

The original contributions presented in this study are included in the article. Further inquiries can be directed to the corresponding author.
